# Validation of a Triplex Pharmacokinetic Assay for Simultaneous Quantitation of HIV-1 Broadly Neutralizing Antibodies PGT121, PGDM1400, and VRC07-523-LS

**DOI:** 10.3389/fimmu.2021.709994

**Published:** 2021-08-24

**Authors:** Martina S. Wesley, Kelvin T. Chiong, Kelly E. Seaton, Christine A. Arocena, Sheetal Sawant, Jonathan Hare, Kasey Hernandez, Michelle Rojas, Jack Heptinstall, David Beaumont, Katherine Crisafi, Joseph Nkolola, Dan H. Barouch, Marcella Sarzotti-Kelsoe, Georgia D. Tomaras, Nicole L. Yates

**Affiliations:** ^1^Center for Human Systems Immunology, Duke University, Durham, NC, United States; ^2^Department of Surgery, Duke University, Durham, NC, United States; ^3^International AIDS Vaccine Initiative (IAVI), Human Immunology Laboratory, Imperial College, London, United Kingdom; ^4^International AIDS Vaccine Initiative (IAVI), New York, NY, United States; ^5^Center for Virology and Vaccine Research, Beth Israel Deaconess Medical Center, Boston, MA, United States; ^6^Ragon Institute of Massachusetts General Hospital (MGH), Massachusetts Institute of Technology (MIT) and Harvard, Cambridge, MA, United States; ^7^Department of Immunology, Duke University, Durham, NC, United States; ^8^Department of Molecular Genetics and Microbiology, Duke University, Durham, NC, United States

**Keywords:** antibody, immunoprophilaxis, HIV - human immunodeficiency virus, pharmacokinetics, validation, broadly neutralizing antibodies

## Abstract

The outcome of the recent Antibody Mediated Prevention (AMP) trials that tested infusion of the broadly neutralizing antibody (bnAb) VRC01 provides proof of concept for blocking infection from sensitive HIV-1 strains. These results also open up the possibility that triple combinations of bnAbs such as PGT121, PGDM1400, as well as long-lasting LS variants such as VRC07-523 LS, have immunoprophylactic potential. PGT121 and PGDM1400 target the HIV-1 V3 and V2 glycan regions of the gp120 envelope protein, respectively, while VRC07-523LS targets the HIV-1 CD4 binding site. These bnAbs demonstrate neutralization potency and complementary breadth of HIV-1 strain coverage. An important clinical trial outcome is the accurate measurement of *in vivo* concentrations of passively infused bnAbs to determine effective doses for therapy and/or prevention. Standardization and validation of this testing method is a key element for clinical studies as is the ability to simultaneously detect multiple bnAbs in a specific manner. Here we report the development of a sensitive, specific, accurate, and precise multiplexed microsphere-based assay that simultaneously quantifies the respective physiological concentrations of passively infused bnAbs in human serum to ultimately define the threshold needed for protection from HIV-1 infection.

## Introduction

The rate of Acquired Immunodeficiency Syndrome (AIDS)-related deaths is not decreasing, despite the existence of highly efficient drugs that suppress Human Immunodeficiency Virus (HIV) replication and provide patients a life expectancy close to that of healthy individuals (1). This is partially due to the lack of sufficient access to antiretroviral therapy (ART) and due to the fact that ART does not eliminate viral reservoirs from HIV-1 infected individuals. Therefore, continuous therapy is needed for a lifetime. Additionally, most currently available ART regimens require daily adherence and have negative side effects, including risk of adverse short- and long-term effects on kidneys, bone density and the cardiovascular system (2–4). Thus, alternate prevention and treatment strategies are needed to increase accessibility and uptake. In particular, effective, long-acting prevention strategies with fewer off-target or other side effects may increase trust and acceptance in communities affected by or at high-risk for HIV-1 acquisition.

Recent studies have shown that passively infused broadly neutralizing monoclonal antibodies (bnAbs) exhibit favorable safety profiles and are promising strategies for therapy and prevention of HIV-1 (5–7). The Antibody Mediated Prevention (AMP) studies substantiated the concept that a bnAb can prevent HIV acquisition (5, 8–10). In addition to prevention of HIV-1 infection, bnAbs are being investigated as an approach to achieve viral control without the use of antiretroviral therapy (11–13). For treatment, as well as for prevention, suitable combinations of antibodies are essential to increase overall breadth and potency of coverage and to prevent the emergence of resistant variants. More than a decade ago, the first bnAbs were successfully isolated from chronically HIV-1 infected individuals (14–16), including VRC01, PGT121 and PGDM1400. PGT121 targets HIV-1 gp120 envelope protein at the base of the V3 glycan loop, PGDM1400 binds to the V1/V2 glycan region (16–18) and VRC01 targets the CD4 binding site. While these are naturally occurring HIV-1 broadly neutralizing antibodies, next generation antibodies have been engineered for increased potency, half-life and ability to target 2 or 3 independent viral sites to achieve better neutralization (19). A good example of this is VRC07-523-LS. VRC07-523-LS is a modified variant of VRC01 and targets the CD4 binding site of the HIV-1 gp120 (20). PGT121, PGDM1400 and VRC07-523-LS in any combination are currently being tested in various clinical trials (ClinicalTrials.gov Identifier NCT02960581, NCT03205917), highlighting the importance of measuring the pharmacokinetics (PK) of more than one antibody simultaneously. These results describe the Triplex PK Assay, a validated method to simultaneously measure the PK of PGT121, PGDM1400 and VRC07-523-LS monoclonal antibody (mAb) concentrations in human serum. This assay utilizes a mixture of three microsphere sets that are each bound to specific anti-idiotype (anti-ID) antibodies to capture either PGT121, PGDM1400 or VRC07-523-LS mAbs. The microsphere mixture is incubated with sample serum and bound mAbs are then detected using a phycoerythrin (PE)-labelled anti-human IgG antibody. Each microsphere set, and therefore, the binding to each mAb, can be distinguished from each other by a Bio-Plex 200 system. It has proven to be sensitive, specific, accurate, and precise in both HIV-1 seronegative and seropositive human serum (Supplementary Material). This method can therefore be applied for analyzing results of both prevention and/or therapeutic human trials and is, hence, an important tool for the effort to end HIV-1 infections and AIDS.

## Material and Methods

### Antibody Capture

**Figure 1:**
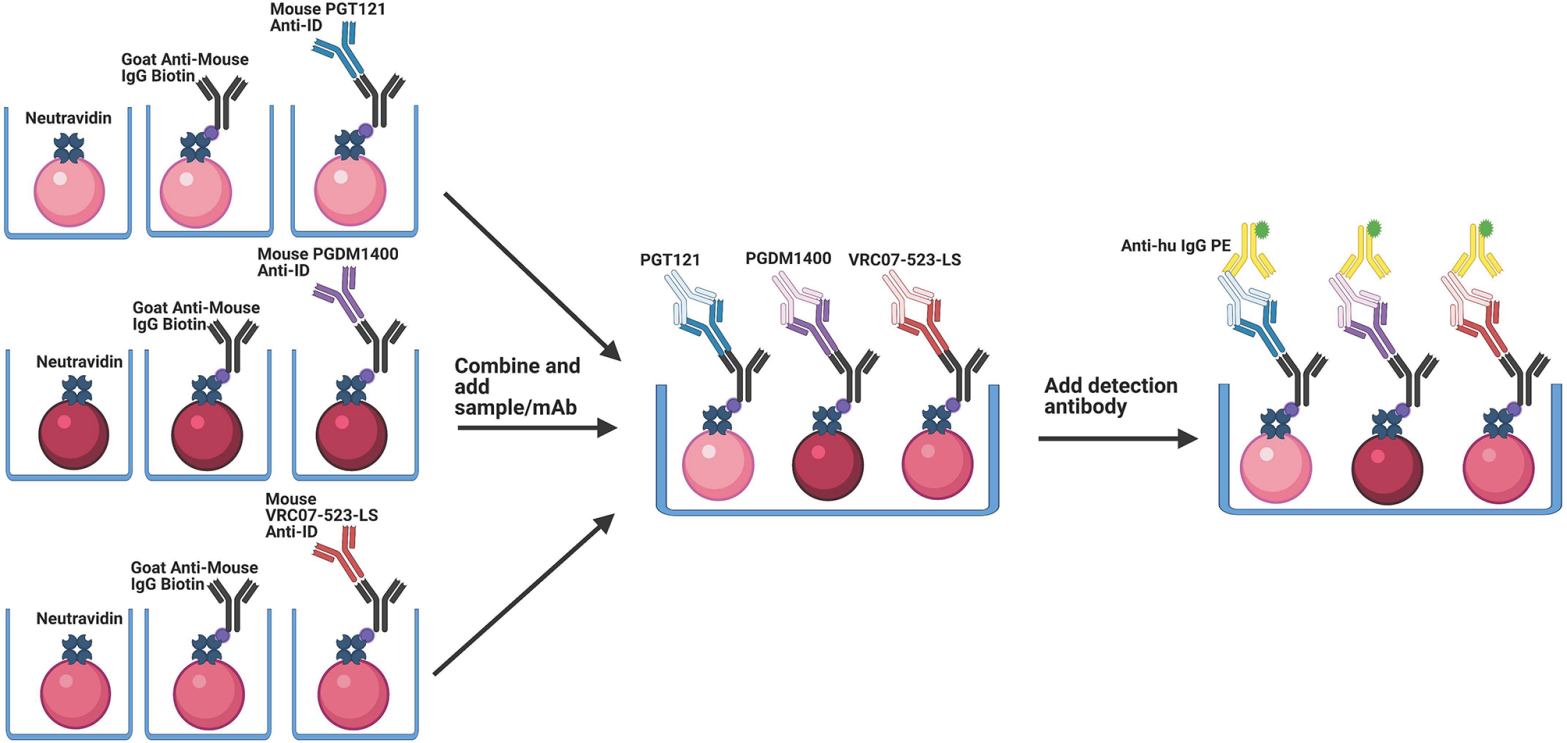
Schematic of the Triplex PK setup. Magnetic microspheres are coupled to NeutrAvidin, which binds to the biotin conjugated goat anti-mouse IgG. This antibody is in an orientation that allows for binding of the anti-ID antibody in the correct orientation to allow for optimal binding of the corresponding mAb. In a multiplex setup, the anti-ID antibodies are bound to the goat anti-mouse IgG Biotin in separate reactions, are washed, and then combined. PGT121, PGDM1400 and VRC07-523-LS mAbs are then added simultaneously into the solution. The microspheres are then washed, and detection antibody is added.

Anti-ID antibodies bind specifically to the antigen binding site, or idiotype, of another antibody. All anti-ID antibodies utilized here are recombinant and generated to bind specifically to their corresponding mAb drug product. Therefore, anti-ID antibodies are important reagents for specific drug development since they can be used to measure free and total drug levels in samples. In the Triplex PK Assay, anti-idiotype antibodies are loaded onto MagPlex microspheres (Luminex Corp, Austin, TX) such that their antigen binding sites are free to bind the antigen binding site of the target antibody drug product. This is done by first binding biotinylated anti-mouse IgG to neutravidin-coupled microspheres, followed by binding to one of the three anti-IDs (**Figure 1**). Each MagPlex microsphere region is labeled with varying concentrations of two different fluorescent dyes, to be individually identified using the Bio-Plex Manager and Bio-Plex 200 Systems (Bio-Rad, Hercules, CA). Covalent coupling of free amines on the side chains of NeutrAvidin (16.65 µg/5x10^6^ microspheres) (ThermoFisher, Waltham, MA) to the intermediate Sulfo-NHS (N-hydroxysulfosuccinimide) ester of activated carboxylated fluorescent MagPlex microspheres was performed according to the xMAP^®^ Antibody Coupling Kit User Manual (Luminex Corp, Austin, TX) (21–24). NeutrAvidin-coupled microspheres were then bound to 5 µg/5x10^6^ microspheres of biotinylated goat anti-mouse IgG antibody, adsorbed against human immunoglobins and pooled sera (SouthernBiotech, Birmingham, AL) for one hour, followed by washing. Goat anti-mouse IgG bound microspheres were then bound to 250 µg/ml of the anti-PGT121-idiotype (Covance, Princeton, NJ, customized and expressed by cell line: IV1737.939.26(A-I0715)) or 25 µg/ml of the anti-PGDM1400-idiotype (Covance, Princeton, NJ, customized and expressed by cell line: IV1919.265.37(A-F0914)) diluted in 500 µl Phosphate Buffered Saline (PBS) for 30 minutes. For detection of VRC07-523-LS mAb, 5 µg/ml of a biotinylated goat anti-mouse IgG Fc antibody, cross- adsorbed against bovine, horse and human serum (ThermoFisher, Waltham, MA) was bound to neutravidin beads for 1 hour, followed by 20 µg/ml anti-VRC07-523-LS-idiotype (5C9)(NIH, Bethesda, MD, Vaccine Research Center, customized and expressed by cell line: CHO DG44) antibody diluted in 500 µl PBS for 30 minutes. Thus, the F(ab) arms are oriented outward to freely bind the paratopes of PGDM1400, PGT121 or VRC07-523-LS (**Figure 1**). Both the capture antibody and anti-ID antibody incubations were done at room temperature (RT) and shaking at 1100rpm. Microspheres were washed and blocked in between incubations with wash buffer (1% Bovine Serum Albumin (w/v)/0.05% Tween-20 (v/v)/0.05% Sodium Azide (w/v) in PBS).

### Triplex PK Assay for HIV-1 bnAbs

The assay was conducted in a 96-well flat bottom plate (Bio-Rad, Hercules, CA). Human sera and controls were diluted in assay diluent (1% Milk Blotto (w/v)/5% Normal Goat Sera (v/v)/0.05% Tween-20 (v/v) in PBS) to the desired dilution factor. Prepared capture microspheres (5000 microspheres/well) were incubated with human sera as unknown samples alongside mAb dilution series of known concentration for generation of standard curves and mAb spiked serum controls (25 µl total volume per well) for 2 hours shaking at 750 rpm and at 22˚C in a temperature controlled incubator. Sample incubation is then followed by 3 washing and blocking steps with 100 µl per well wash buffer using a magnetic separator (Luminex Corp, Austin, TX). Subsequently, 25 µl per well of a goat anti-human IgG detection antibody conjugated to phycoerythrin (PE) at 2 µg/ml for 30 minutes (SouthernBiotech, Birmingham, AL) was used to detect bound PGDM1400, PGT121 and VRC07-523-LS. Known amounts of purified PGT121 IgG mAb (0.5 µg/ml, 2-fold, 14 serial dilutions) (Catalent, Somerset, NJ), PGDM1400 IgG mAb (0.1 µg/ml, 2-fold, 14 serial dilutions) (Catalent, Somerset, NJ) and VRC07-523-LS IgG mAb (1 µg/ml, 3-fold, 14 serial dilutions) (NIH, Bethesda, MD) were co-titrated as a standard curves on every assay plate to quantify PGT121 IgG, PGDM1400 IgG and VRC07-523-LS IgG present in human serum using fluorescence intensity (FI) readouts with the unit of median fluorescence intensity (MFI). All controls, samples, standards, and blanks were assayed as a duplicate, and the mean value was reported. The blank well (background, referred to as Bkgd) is subtracted from the FI and the resultant value was reported as FI-Bkgd. Additionally, PGDM1400 IgG mAb, PGT121 IgG mAb and VRC07-523-LS IgG mAbs were spiked into 1:100 normal human reference serum (NHS) at 5 specific known concentrations (1. PGDM1400 starting at 0.04 µg/ml, 4-fold, for 5 serial dilutions; 2. PGT121 starting at 0.08 µg/ml, 2-fold, for 4 serial dilutions plus a fifth concentration of 0.002 µg/ml; and 3. VRC07-523-LS starting at 0.002 µg/ml, 2-fold, for 5 serial dilutions) and were used as antibody-specific controls for standard curve accuracy. Negative controls and blank (uncoupled) microspheres were included in each assay to ensure specificity. Assay plates were read using Bio-Plex Manager and Bio-Plex 200 Systems (Bio-Rad, Hercules, CA). The Bio-Plex 200 System detects the fluorescent dyes (microsphere region) within the each MagPlex microsphere region and then quantifies the associated PE signal intensities from the detection antibody. The level of bound mAbs is identified by the intensity of the PE (reporter signal) in FI-Bkgd. This enables the quantification of all three mAbs simultaneously using Bio-Plex Manager software.

### Specimens

Human sera were obtained from HIV seronegative and seropositive individuals, with Institutional Review Board (IRB) approval. Both seronegative and seropositive samples were assessed to test assay parameters in the sample-specific matrix, with applications for both HIV-1 prevention and therapeutic studies. 132 HIV seronegative human sera were obtained from BioIVT, Westbury, NY (formerly Bioreclamation), and 66 HIV seropositive samples were obtained from the University of Washington Center For AIDS Research (Seattle, WA). An additional 17 samples were obtained from group 3B in the CAVD study Barouch 693 (IAVI T002, ClinicalTrials.gov Identifier NCT03205917), from seropositive study participants off ART and infused with PGDM1400 and/or PGT121.

### Quality Control/Quality Assurance and Data Management

Quality control (QC) acceptance criteria for Triplex PK assay include but are not limited to: 1) FI-Bkgd readout must have a percentage of coefficient of variation (%CV) between replicates < 20% for each dilution in the titration series and < 15% for a sample that is assayed at a single dilution when fluorescence- background (FI-Bkgd)>100, 2) Three out of five spiked controls must have a recovery between 70 -130% of their input concentration, and 3) the positive control antibody titer, defined as the half maximal effective concentration (EC50) from a five parameter logistic curve fit (5PL) and the highest FI-Bkgd in the positive control standard curve must be within three standard deviations of the historical mean as tracked with Levey-Jennings charts [Portal, Labkey, Seattle, WA (25)]. Guide sets for Levey-Jennings charts that enable tracking of positive controls to historical data were established from >10 standardized assays. Experiments were performed in a Good Clinical Laboratory Practice (GCLP) compliant laboratory with oversight by the Quality assurance for Duke Vaccine Immunogenicity Programs (QADVIP). Assay documentation was recorded and stored in an Electronic Laboratory Notebook (ELN) (Agilent Technologies, Santa Clara, CA). Raw experimental data and analyses are securely stored with an audit trail through Electronic Content Manager (ECM) (Agilent Technologies, Santa Clara, CA).

### Analysis

Validation described herein was performed in accordance with the FDA document “Guidance for Industry: Bioanalytical Method Validation, May 2018” and the ICH Tripartite Guideline “Validation of Analytical Procedures: Text and Methodology. Q2(R1)” (26, 27). Qualification of the method for HIV-1 seropositive serum followed the same aspects and parameters, with a smaller sample size than for validation. Should a need for validation of this method using seropositive serum be deemed necessary, established cut points from qualification will be used for validation. The parameters tested and reported here are Accuracy, Specificity, Positivity, Limit of Detection and Quantitation, and Precision. Range and linearity testing were not specifically tested since samples can be diluted to fall within the quantifiable range of the assay, and samples are screened at the beginning of the trial to determine optimal dilution factor. This is a newly developed assay and is not performed in another laboratory, so reproducibility could not be tested. The recommended sample size for establishing limit of detection (LOD) and lower limit of quantitation (LLOQ) is 60 (28). Therefore, at least 66 (110% of required minimum) samples were tested because it is anticipated that some samples might not pass the QC criteria and are, therefore, not included in the calculations. 5PL curve fit for the standard curves as well as the equation for EC50 calculation were generated with Bio-Plex Manager (Bio-Rad). For validation in seronegative samples and to enhance the significance in establishing the background cutoffs (LOD and LLOQ), the FI-Bkgd of two samples sets (66 each, 132 seronegative samples in total) were combined to calculate the 95^th^ percentile FI-Bkgd. The LOD in FI-Bkgd (mean + 3.3 standard deviations of the FI-Bkgd from these samples) was plugged into the 5PL equation generated by Bio-Plex Manager from the PGDM1400 IgG, PGT121 IgG or VRC07-523-LS IgG standard curve to obtain the concentration for the LOD. LLOQ was calculated in a similar way, except that the mean + 10 standard deviations of the FI-Bkgd from these samples was be used. The LLOQ in FI-Bkgd was then also plugged into the 5PL equation from respective standard curve to calculate the concentrations of the LLOQ. Alternatively, LLOQ can be determined as the lowest accurately-recovered concentration on the standard curve that it was above the LOD. Microsoft Excel was used to calculate %CV (standard deviation/mean *100) between curves for repeatability and intermediate precision, Levey-Jennings plots, and calculating concentration for LOD and LLOQ with the 5PL curve. Data processing for automated QC report was performed on a secure and validated data repository [Portal, Labkey (25)]. SAS software, Version 9.4 of the SAS System for Windows, Copyright ^©^ [2002-2012] by SAS Institute Inc., Cary, NC, USA, was used for generating histograms. R Core Team (2018). R: A language and environment for statistical computing, R Foundation for Statistical Computing, Vienna, Austria. URL https://www.R-project.org/ and package ggplot2 was used for generating plots for positivity.

## Results

### Specificity

The specificity of an analytical method is the capability of assessing the analyte in the presence of other components, which may have a positive or negative effect on the resulting values. This can also be defined as non-specific background. Components that may be expected are impurities, degradation products and/or sample matrix. Whether the presence of one mAb affects the background and specificity of the detection of the other mAb concentrations are of particular interest for co-infusion studies. The goal of this experiment was to assess how the three mAbs influence each other or the output values of this method.

#### Non-Specific Background Activity of HIV-1 Seronegative Human Serum

**Figure 2:**
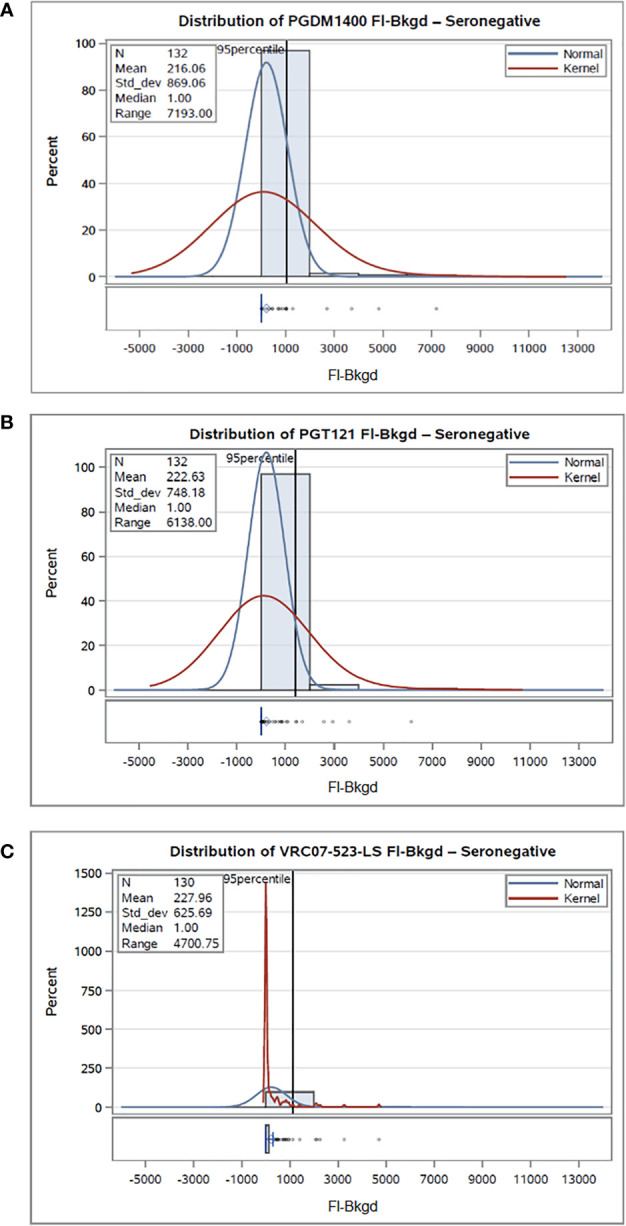
Non-specific background activity of HIV-1 seronegative human serum. 132 HIV-1 seronegative samples were tested at 1:100 in the PGDM1400/PGT121/VRC07-523-LS PK BAMA, and the distribution of binding to the PGDM1400 anti-ID **(A)**, PGT121 anti-ID **(B)**, and VRC07-523-LS anti-ID **(C)** was plotted. Normal and kernel density estimates are used to visualize the data distribution. Normal shows the fit of a normal distribution to the FI-Bkgd measurements, and a superimposed fitted density curve on the histogram. Kernel density estimates on histograms help smoothen the data distribution and help visualize the non-normal data distribution and patterns.

To assess non-specific background activity in human serum, 132 HIV-1 seronegative sera (2 subsets of each 66 samples) were diluted at 1:100 and tested for non-specific background binding. Seropositive sera were also tested, and data is shown in Supplementary Material. The FI-Bkgd values (excluding samples that did not meet QC acceptance criteria) were used to calculate a mAb specific positivity threshold. The 95^th^ percentile is an established method for antigen specific cutoff determination from baseline or seronegative status samples and is commonly used for immunoassays (29, 30). The positivity cutoff was calculated as the 95^th^ percentile of the mAb and sample set specific response from the tested samples (**Table 1** and **Supplementary Table 1**). Positivity cutoffs for PGDM1400, PGT121 and VRC07-523-LS in HIV-1 seronegative serum are as low as 1049 FI-Bkgd, 1419 FI-Bkgd and 1126 FI-Bkgd, respectively (**Table 1**). **Figure 2** shows the FI-Bkgd distribution of binding to anti-IDs for PGDM1400 (**Figure 2A**), PGT121 (**Figure 2B**) and VRC07-523-LS (**Figure 2C**) in seronegative (**Figures 2A–C** **)** and seropositive (**Supplementary Figures 1A–C** **)** human serum. Thus, these results demonstrate a sensitive assay with low positivity cutoffs.

**Table 1:** Limit of detection and quantification in HIV-1 seronegative human serum.

mAb	Serostatus	95th percentile (FI-Bkgd)	LOD ng/ml	Physiological LOD µg/ml	LLOQ ng/ml (mean +10 stdev)	Physiological LLOQ µg/ml	ULOQ ng/ml (highest concentration with accurate recovery)	Physiological ULOQ µg/ml	LLOQ ng/ml (lowest accurate point above LOD)	Physiological LLOQ µg/ml
***PGDM1400***	Seronegative	1049	0.26	0.026	0.76	0.076	50	5	0.391	0.039
***PGT121***	Seronegative	1419	0.91	0.091	2.6	0.26	250	25	0.977	0.098
***VRC07-523-LS***	Seronegative	1126	0.14	0.014	0.41	0.041	37	3.7	0.152	0.098

LOD, LLOQ and ULOQ as well as the corresponding physiological concentrations observed in the PGDM1400/PGT121/VRC07-523-LS Triplex PK for HIV-1 seronegative human serum.

#### Positivity

**Figure 3:**
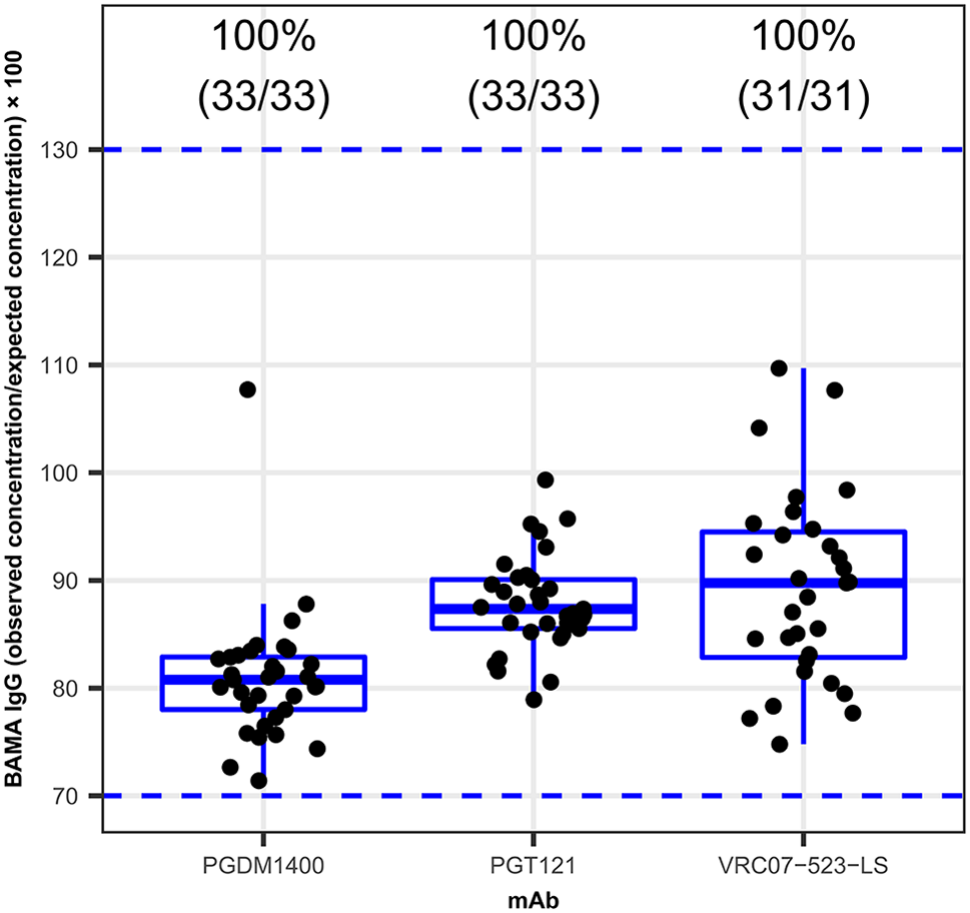
Positivity of the PGDM1400, PGT121 and VRC07-523-LS Triplex PK. 33 HIV-1 seronegative serum samples were spiked with specific concentrations of the PGDM1400, PGT121, and VRC07-523-LS. Percent recovery, calculated as (observed concentration ÷ expected concentration) × 100, for PGMD1400, PGT121 and VRC07-523-LS, respectively is shown on y-axis. The blue dashed lines at levels 70 and 130, show preset acceptance criteria for percent recovery [70-130%]. Numbers on top of each box plot show the percentage and counts (number of samples that met the present acceptance criteria/total number of samples) of samples with a percent recovery between 70-130%. 100% of the samples, for all three bnAbs tested here, had acceptable percent recovery.

To determine if the reported method could detect and quantify accurate amounts of PGDM1400, PGT121, and VRC07-523-LS mAb in 33 HIV-1 seronegative serum samples, commercially available samples were spiked with known amounts of each mAb (0.006 µg/ml of PGDM1400, 0.020 µg/ml of PGT121, and 0.003 µg/ml of VRC07-523-LS). These concentrations were selected since they are within the limits of accurate recovery (70-130%) for the respective mAb and above the calculated limit of detection. Percent recovery was calculated using following formula: (observed concentration/expected concentration)*100. Of all included samples, 100% of seronegative samples (**Figure 3**) and at least 80% of seropositive (**Supplementary Figures 2A–C** **)** samples had an observed concentration that was within 2-fold of the expected concentration.

### Accuracy

Accuracy of an analytical method is expressed by the degree to which the value found conforms with a particular value, which is either the accepted conventional true values or an accepted reference value. It also demonstrates how reliably a method can produce true values, which is an essential parameter for the pharmacokinetic measurement of passively-infused bnAbs. To determine the accuracy of antibody concentration detection, or antibody recovery, in the sample matrix, PGDM1400 IgG, PGT121 IgG and VRC07-523-LS IgG mAbs were co-titrated into assay diluent as well as into pooled HIV-1 seronegative sera diluted at 1:100 in assay diluent. Additionally, as an additional test of antibody recovery to ensure the accuracy of the standard curve, PGDM1400, PGT121 and VRC07-523-LS mAbs at several known concentrations (low, medium, high) were spiked into assay diluent and into 30 pooled HIV-1 seronegative human serum samples, diluted 1:100 diluted in assay diluent. The known concentrations for each mAb spiked controls were as follows: 1. PGDM1400 starting at 0.04 µg/ml, 4-fold, for 5 serial dilutions; 2. PGT121 starting at 0.08 µg/ml, 2-fold, for 4 serial dilutions plus a fifth concentration of 0.002 µg/ml; and 3. VRC07-523-LS starting at 0.002 µg/ml, 2-fold, for 5 serial dilutions. A PGDM1400/PGT121/VRC07-523-LS IgG co-titrated standard curve was assayed on the same plate to determine observed concentration of the spiked serum samples. It was also critical to determine if the bnAbs tested had any cross-reactivity to anti-idiotypes of mis-matched specificity. Therefore, a co-titrated PGDM1400/PGT121 and a VRC07-523-LS standard curve, both from validated assays, were compared to the co-titrated PGMD1400/PGT121/VRC07-523-LS standard curve. All three curves were titrated in 1:100 NHS diluted in assay diluent and FI-Bkgd as well as percent recovery were compared. Observed concentrations and percent recovery (observed concentration/expected concentration x 100) were calculated using the Bio-Plex Manager software. Additionally, to determine if each bnAb demonstrated cross-reactivity to the un-matched anti-ID antibodies, each bnAb was solely spiked into assay diluent at defined concentrations as mentioned above and incubated with a microsphere mixture containing all three anti-ID antibodies.

**Figure 4:**
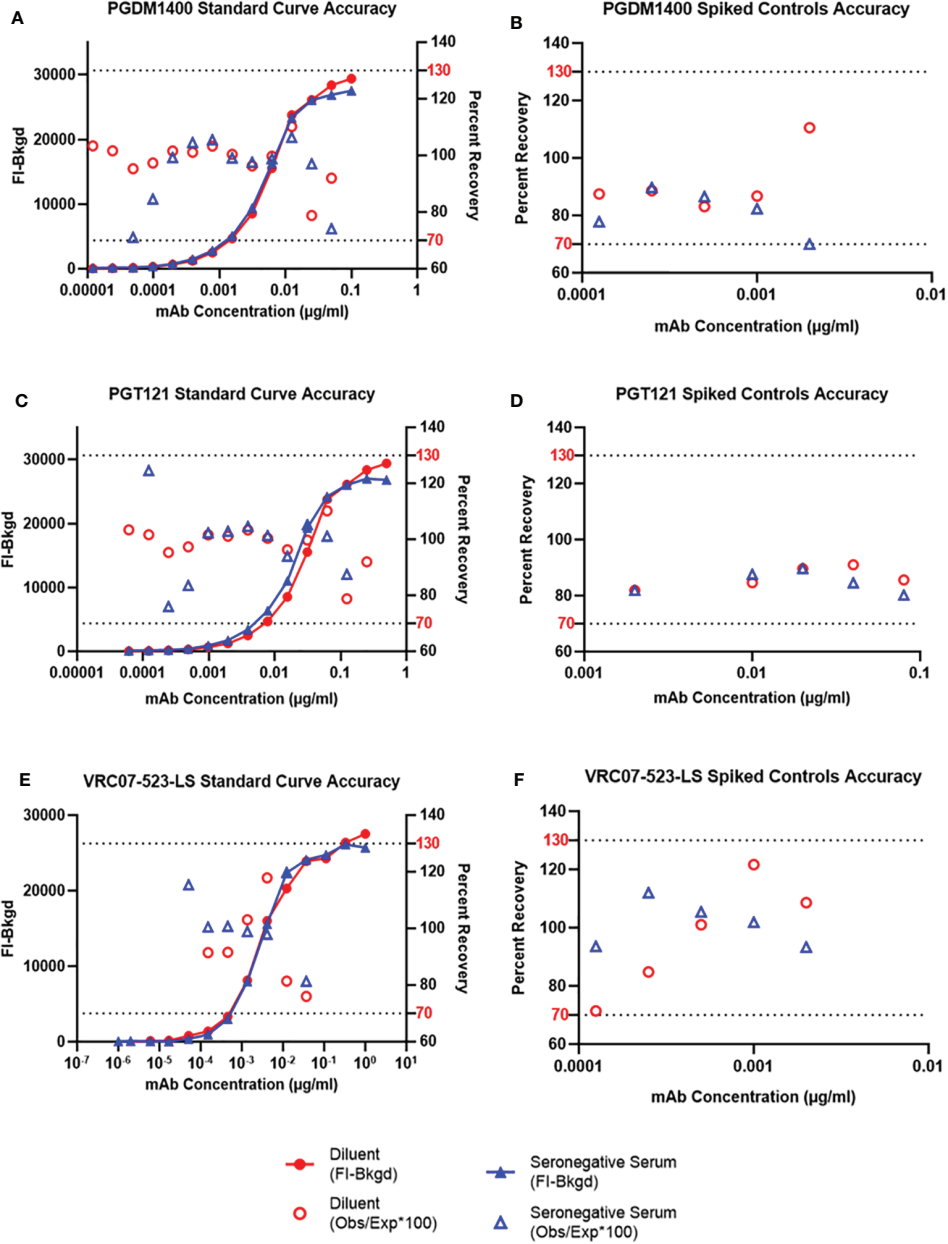
Accuracy of Standard Curves and Spiked Sample Recovery. PGDM1400 IgG **(A)**, PGT121 IgG **(C)** and VRC07-523-LS IgG **(E)** mAbs were titrated in diluent (red), in pooled 1:100 diluted HIV-1 seronegative human serum. PGDM1400 IgG **(B)**, PGT121 IgG **(D)** and VRC07-523-LS IgG **(F)** mAbs were spiked in pooled 1:100 diluted seronegative serum at 5 different concentrations. Observed concentration for each point in the curve was calculated and used to determine accuracy of recovery of each mAb. The dotted lines denote the upper (130% of the expected concentration) and lower (70% of the expected concentration) limits of acceptable percent recovery.

**Figure 5:**
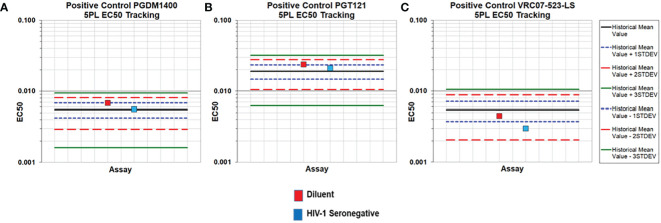
Accuracy of the PGDM1400/PGT121/VRC07-523-LS Triplex PK standard curves in diluent and HIV-1 seronegative human serum. PGDM1400 **(A)**, PGT121 **(B)** and VRC07-523-LS **(C)** were co-titrated in assay diluent and 1:100 HIV-1 seronegative serum diluted in assay diluent. 5PL EC50 values of each mAb fell within 3 standard deviations of the historical mean (solid black line).

**Figure 6:**
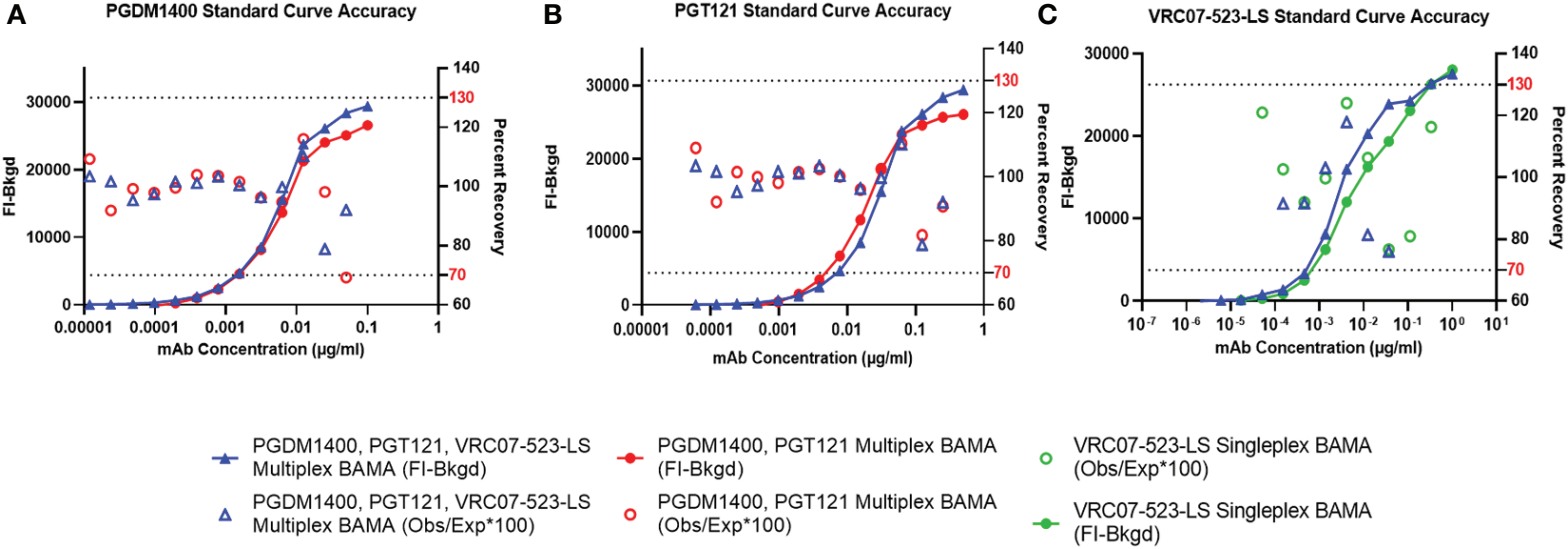
Accuracy of multiplexed PGDM1400, PGT121 and VRC07-523-LS. Co-titrated PGDM1400 **(A)** PGT121 **(B)** as well as a VRC07-523-LS **(C)** standard curve (all from validated assays) are compared to the co-titrated PGMD1400/PGT121/VRC07-523-LS standard curve. All three curves were titrated in 1:100 NHS diluted in assay diluent and FI-Bkgd as well as percent recovery were compared. Curves and percent recovery are comparable indicating that VRC07-523-LS does not bind to any other anti-ID other than the one specific to VRC07-523-LS.

**Figure 7:**
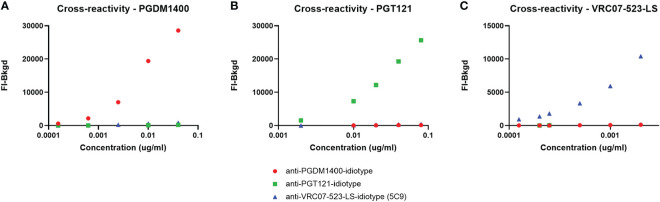
Lack of Cross-reactivity and competition between PGDM1400, PGT121 and VRC07-523-LS and their respective anti-ID antibodies. PGDM1400 **(A)**, PGT121 **(B)** and VRC07-523-LS **(C)** were each titrated in assay diluent and incubated with a microsphere mixture containing all three anti-ID antibodies. Binding can only be observed between specific mAb and anti-ID antibodies, indicating that the anti-ID’s are highly specific and no cross-reactivity can be expected.

The FI-Bkgd observed for the co-titrated PGDM1400/PGT121/VRC07-523-LS standard curves in assay diluent were comparable at most points to those observed in pooled HIV-1 seronegative (**Figure 4**) and seropositive serum (**Supplementary Figure 3**). At least 6 and 7 (for VRC07-523-LS) or 13 and 11 (for PGDM1400 and PGT121) consecutive points had accurate recovery within the range of 70-130% for the co-titrated standard curve in assay diluent and in HIV-1 seronegative serum, respectively (**Figures 4A, C, E** **)**. These results meet the defined validation criteria, which required at least 5 consecutive points in each titration curve to have an observed concentration that is between 70 and 130% of the expected concentration. Recovery for each spiked concentration in pooled HIV-1 seronegative serum was within the accurate recovery range of 70-130% (**Figures 4B, D, F** **).** The 5PL (5 parameter logistic) EC50 (Effective Concentration-50) values of PGDM1400/PGT121/VRC07-523-LS standard curve in assay diluent, HIV-1 seronegative and seropositive serum are within the mean +/- 3 standard deviations criteria established for tracking in Levey-Jennings (**Figure 5** and **Supplementary Figure 4**). Results for FI-Bkgd and percent recovery for the PGDM1400/PGT121 and VRC07-523-LS standard curves and comparable to the co-titrated PGDM1400/PGT121/VRC07-523-LS standard curve, demonstrating that VR07-523-LS did not bind to the PGT121 or PGDM1400 anti-ID antibodies and no cross-reactivity was observed **(** **Figure 6** **)**. Binding was only observed between specific mAb and anti-ID antibodies, indicating that they are highly specific (**Figure 7** **).** These results demonstrate that the co-titrated PGDM1400/PGT121/VRC07-523-LS standard curves in assay diluent, HIV-1 seronegative as well as seropositive serum can accurately and simultaneously quantify each mAb concentration respectively, in the serum matrix diluted to be within the linear range of the assay.

### Limits of Detection and Quantitation

The limit of detection (LOD) is the lowest concentration of an analyte that can be detected but not necessarily quantified, while the lower limit of quantitation (LLOQ) is the lowest concentration that can be quantified with acceptable precision. The upper limit of quantitation (ULOQ) is the highest concentration at which each mAb can be consistently and accurately recovered. The level of background binding and therefore, the LOD (mean + 3.3 standard deviations of FI-Bkgd) and LLOQ (mean + 10 standard deviations of FI-Bkgd), may be highly dependent on the sample set tested, therefore study-specific LOD/LLOQ should be determined using baseline samples, population-specific or drug naïve samples. For validation in seronegative samples and to enhance the significance in establishing the background cutoffs (LOD and LLOQ), the FI-Bkgd of two samples sets (66 each, 132 seronegative samples in total) were combined to calculate the 95^th^ percentile. The physiological LOD and LLOQ are the values multiplied by the dilution factor and therefore, corresponding to the concentration in the sample before diluting for the assay.

PGDM1400, PGT121 and VRC07-523-LS were co-titrated in 30 pooled HIV-1 seronegative serum samples diluted at 1:100 in assay diluent for the standard curve. 132 HIV-1 seronegative samples were diluted at 1:100. All samples that fell below the 95^th^ percentile cutoff (**Table 1**) were used for the analysis. The LOD, LLOQ (using both calculations mentioned above), ULOQ and their corresponding physiological concentrations for each mAb in HIV-1 seronegative human serum are summarized in **Table 1**, and **Supplementary Table 1** for HIV-1 seropositive human serum.

### Precision

Precision is a measurement of the random error or degree of scatter between repeated measurements, which are repeated under the same conditions. Precision may be considered at two levels: repeatability and intermediate precision.

#### Repeatability

**Figure 8:**
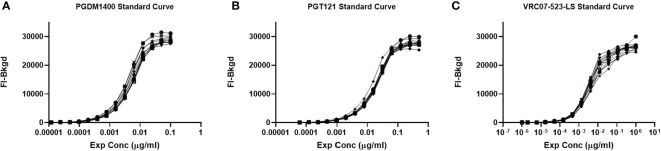
Repeatability of the PGDM1400/PGT121/VRC07-523-LS standard curve. PGDM1400, PGT121 and VRC07-523-LS were co-titrated in 1:100 NHS in assay diluent for a total of 12 repeats by the same operator. Curves for PGDM1400 **(A)**, PGT121 **(B)** and VRC07-523-LS **(C)** are graphed to demonstrate the repeatability of this assay.

Repeatability testing was performed to assess the variation in the PGDM1400/PGT121/VRC07-523-LS co-titrated standard curve when tested repeatedly. 12 repeats for each 12 dilutions of the standard curve were analyzed and performed by the same operator (**Figure 8**). The %CV between each replicate was less than 18% for all dilutions within the linear range of the curve for each mAb (**Table 2**). Thus, this assay passes the preset validation criteria, which is defined that the %CV between each replicate has to be below 30% within the accurate range of the standard curve.

**Table 2:** Repeatability of PGDM1400/PGT121/VRC07-523-LS standard curve.

mAb	Conc (μg/ml)	Mean FI-Bkgd	%CV (12 curves)
***PGDM1400***	**0.1**	**28526.25**	**3.92**
	**0.05**	**28256.63**	**4.24**
	**0.025**	**26743.50**	**5.74**
	**0.0125**	**24070.25**	**8.12**
	**0.00625**	**16543.88**	**13.69**
	**0.003125**	**9521.50**	**15.43**
	**0.001563**	**5346.25**	**17.43**
	**0.000781**	**2826.75**	**17.70**
	**0.000391**	**1519.00**	**17.22**
	0.000195	746.38	19.99
	0.000098	360.25	22.11
	0.000049	180.13	27.32
	0.000024	157.38	28.16
	0.000012	85.50	25.79
***PGT121***	**0.5**	**27582.50**	**3.90**
	**0.25**	**27293.38**	**3.94**
	**0.125**	**26578.63**	**3.16**
	**0.0625**	**24460.25**	**2.84**
	**0.03125**	**18272.13**	**4.46**
	**0.015625**	**11092.50**	**4.59**
	**0.007813**	**6342.63**	**6.82**
	**0.003906**	**3342.13**	**5.66**
	**0.001953**	**1720.25**	**10.49**
	0.000977	843.13	7.20
	0.000488	393.75	13.52
	0.000244	188.13	23.56
	0.000122	144.50	25.80
	0.000061	78.38	29.48
***VRC07-523-LS***	**1**	**26137.25**	**5.03**
	**0.333333**	**25989.13**	**3.75**
	**0.111111**	**24656.63**	**5.46**
	**0.037037**	**22778.63**	**8.45**
	**0.012346**	**20545.50**	**11.35**
	**0.004115**	**13522.25**	**11.35**
	**0.001372**	**6874.38**	**11.24**
	**0.000457**	**2803.50**	**13.58**
	0.000152	919.63	16.44
	0.000051	372.00	28.22
	0.000017	131.38	65.48
	0.000006	50.75	173.37
	0.000002	84.25	66.03
	0.000001	48.38	157.62

The table shows the average binding (Mean FI-Bkgd) of each of the 12 titrated curves at each dilution as well as the %CV, which was less than 18% at each dilution within the linear range of the curves. Values in bold are above the FI-Bkgd LOD for the respective mAb and curve.

#### Intermediate Precision

**Figure 9:**
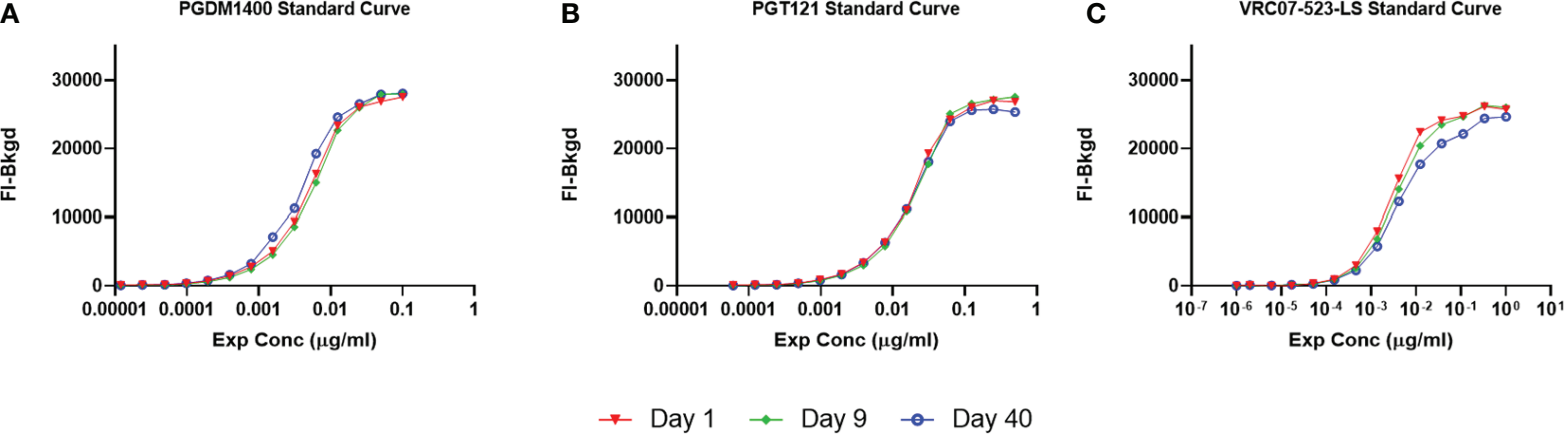
Intermediate Precision of the PGDM1400/PGT121/VRC07-523-LS standard curve. PGDM1400, PGT121 and VRC07-523-LS were co-titrated in 1:100 NHS in assay diluent by the same operator over a timeframe of 40 days. Curves for PGDM1400 **(A)**, PGT121 **(B)** and VRC07-523-LS **(C)** are graphed to demonstrate the intermediate precision of this assay.

Intermediate precision assesses the variation across days, assay operators and/or equipment (27). To determine the intra-operator variability over 40 days, 3 assays including a PGDM1400/PGT121/VRC07-523-LS standard curve were performed. Resulting FI-Bkgd and observed concentrations were compared (**Figure 9** and **Table 3**). Similar to the parameters for repeatability, the %CV of each point of the standard curve within the linear range was less than 30%. Thus, this method demonstrates intermediate intra-operator precision over the time course of 40 days due to the %CV being a maximum of 14% for each point within the linear range (**Table 3**).

**Table 3:** Intermediate precision of PGDM1400/PGT121/VRC07-523-LS standard curve.

mAb	Conc (μg/ml)	Mean FI-Bkgd	%CV (3 curves)
***PGDM1400***	**0.1**	**28018.88**	**1.79**
	**0.05**	**27914.25**	**2.13**
	**0.025**	**26289.13**	**1.38**
	**0.0125**	**23187.63**	**3.55**
	**0.00625**	**15701.00**	**11.94**
	**0.003125**	**9146.88**	**12.46**
	**0.001563**	**5118.38**	**20.86**
	**0.000781**	**2746.13**	**12.31**
	**0.000391**	**1424.63**	**12.34**
	0.000195	726.00	10.88
	0.000098	373.75	15.55
	0.000049	180.13	26.74
	0.000024	166.88	4.53
	0.000012	103.63	18.23
***PGT121***	**0.5**	**27042.75**	**3.67**
	**0.25**	**27073.38**	**2.77**
	**0.125**	**26298.25**	**3.13**
	**0.0625**	**24228.63**	**1.99**
	**0.03125**	**18661.75**	**4.51**
	**0.015625**	**11092.50**	**3.39**
	**0.007813**	**6351.63**	**8.40**
	**0.003906**	**3392.25**	**7.07**
	**0.001953**	**1719.13**	**8.48**
	0.000977	877.63	9.35
	0.000488	398.75	15.16
	0.000244	201.50	26.83
	0.000122	160.00	13.07
	0.000061	84.75	33.69
***VRC07-523-LS***	**1**	**25855.00**	**5.03**
	**0.333333**	**26093.88**	**3.75**
	**0.111111**	**24698.63**	**5.46**
	**0.037037**	**23454.38**	**8.45**
	**0.012346**	**21074.75**	**11.35**
	**0.004115**	**14348.63**	**11.35**
	**0.001372**	**7102.88**	**11.24**
	**0.000457**	**2736.75**	**13.58**
	0.000152	940.63	16.44
	0.000051	341.63	28.22
	0.000017	173.75	65.48
	0.000006	50.75	173.37
	0.000002	120.50	66.03
	0.000001	48.38	157.62

The table shows the average binding (Mean FI-Bkgd) of each standard curve performed by the same operator over the time course of 40 days at each dilution as well as the %CV, which was less than 14% at each dilution within the linear range of the curves. Values in bold are above the LOD in FI-Bkgd for the respective mAb and curve.

## Discussion

The PK method for the simultaneous detection of three bnAbs was validated for HIV-1 seronegative human serum as well as qualified for HIV-1 seropositive human serum for the measurement of antibody concentrations in human clinical trials. Results demonstrated that the PGDM1400/PGT121/VRC07-523-LS PK BAMA is specific, accurate, sensitive, precise, and capable for repeatable simultaneous quantitation of three bnAbs, namely PGT121, PGDM1400, and VRC07-523-LS. This innovative Triplex immunoassay reported here measures the pharmacokinetics of PGDM1400, PGT121 and VRC07-523-LS mAbs simultaneously using PGDM1400-, PGT121- and VRC-07-523-LS-specific anti-idiotype antibodies. This assay accurately quantified each mAb in HIV-1 seronegative specimens at physiological concentrations in serum starting at 0.026 µg/mL, 0.091 µg/mL, and 0.014 µg/mL for PGDM1400, PGT121 and VRC07-523-LS, respectively. This was similar to the LOD of PGDM1400, PGT121 and VRC07-523-LS in HIV-1 seropositive human serum starting at physiological concentrations of 0.032 µg/mL, 0.098 µg/mL, and 0.021 µg/mL, respectively. The upper limits of quantitation in diluted serum for PGDM1400, PGT121 and VRC07-523-LS were 5 µg/mL, 25 µg/mL, and 3.7 µg/mL, respectively, and highly concentrated serum samples can be diluted to fall within the upper and lower quantification limits. Other common parameters like range and linearity were not tested for this report since samples can be diluted to fall within the quantifiable range of the assay. Therefore, samples for a new study will be tested in the beginning of the trial to determine the optimal dilution factor. This assay can not only be used to quantify bnAb concentration after infusion for HIV prevention studies, but also for therapeutic studies in HIV-infected subjects. There is a critical need for reliable methods to analyze the pharmacokinetics of mAbs in the human body, especially with increased abilities to engineer next generation antibodies as well as to verify pharmacokinetic modelling approaches *in vivo* (31, 32). A reliable method is the enzyme-linked immunosorbent assay (ELISA), which has been utilized in most PK studies in the past (12, 13, 17, 33–35). However, traditional ELISA protocols can only assay one analyte at a time. The PK BAMA is a microsphere-based assay that can be customized to assay up to 50 analytes simultaneously and is readily adapted to new bnAbs as well as new bnAb combinations. The simultaneous measurement of multiple bnAbs is an efficient tool to inform the selection of the most promising bnAb combinations for HIV-1 prevention and therapeutics strategies.

## Data Availability Statement

The original contributions presented in the study are included in the article/**Supplementary Material**. Further inquiries can be directed to the corresponding author.

## Ethics Statement

The studies involving human participants were reviewed and approved by Duke IRB. The patients/participants provided their written informed consent to participate in this study.

## Author Contributions

MW contributed to the design of the work, performed experiments, analyzed and interpreted data, and helped with writing the manuscript. KTC, CA, KH, and MR contributed to the conception/design of the work, performed experiments, and analyzed and interpreted data. KS and JHe contributed to the conception/design of the work, analysis and interpretation of data, and revising the manuscript. SS and DB contributed to data analysis and interpretation. JHa, KC, MS-K, and GT contributed to design of work and data interpretation and manuscript revision. JN and DHB contributed to conception and design of the work. NY contributed to conception/design of the work, analysis and interpretation of data, and writing and revising the manuscript. All authors critically reviewed the manuscript for intellectual content and agree to be accountable for all aspects of the work. All authors contributed to the article and approved the submitted version.

## Funding

This work was supported by the Bill & Melinda Gates Foundation, Collaboration for AIDS Vaccine Discovery, Vaccine Immune Monitoring Center (CAVIMC) (OPP1146996); and the National Institutes of Health (NIH), National Institute of Allergy and Infectious Diseases (NIAID, Duke Center for AIDS Research (CFAR) (P30 AI064518) and HIV Vaccine Trials Network Laboratory Program (NIH U01 AI068618).

## Conflict of Interest

The authors declare that the research was conducted in the absence of any commercial or financial relationships that could be construed as a potential conflict of interest.

## Publisher’s Note

All claims expressed in this article are solely those of the authors and do not necessarily represent those of their affiliated organizations, or those of the publisher, the editors and the reviewers. Any product that may be evaluated in this article, or claim that may be made by its manufacturer, is not guaranteed or endorsed by the publisher.

## References

[1] Marcus JL Leyden WA Alexeeff SE Anderson AN Hechter RC Hu H. Comparison of Overall and Comorbidity-Free Life Expectancy Between Insured Adults With and Without HIV Infection, 2000-2016. JAMA Network Open (2020) 3(6):e207954–e. 10.1001/jamanetworkopen.2020.7954 PMC7296391 32539152

[2] Schouten J Wit FW Stolte IG Kootstra NA van der Valk M Geerlings SE. Cross-Sectional Comparison of the Prevalence of Age-Associated Comorbidities and Their Risk Factors Between HIV-Infected and Uninfected Individuals: The AGEhIV Cohort Study. Clin Infect Dis (2014) 59(12):1787–97. 10.1093/cid/ciu701 25182245

[3] Rasmussen LD May MT Kronborg G Larsen CS Pedersen C Gerstoft J. Time Trends for Risk of Severe Age-Related Diseases in Individuals With and Without HIV Infection in Denmark: A Nationwide Population-Based Cohort Study. Lancet HIV (2015) 2(7):e288–98. 10.1016/S2352-3018(15)00077-6 26423253

[4] Hoy JF Grund B Roediger M Schwartz AV Shepherd J Avihingsanon A. Immediate Initiation of Antiretroviral Therapy for HIV Infection Accelerates Bone Loss Relative to Deferring Therapy: Findings From the START Bone Mineral Density Substudy, A Randomized Trial. J Bone Mineral Res (2017) 32(9):1945–55. 10.1002/jbmr.3183 PMC5555813 28650589

[5] Gilbert PB Juraska M deCamp AC Karuna S Edupuganti S Mgodi N. Basis and Statistical Design of the Passive HIV-1 Antibody Mediated Prevention (AMP) Test-Of-Concept Efficacy Trials. Stat Commun Infect Dis (2017) 9(1):20160001. 10.1515/scid-2016-0001 29218117 PMC5714515

[6] Ledgerwood JE Coates EE Yamshchikov G Saunders JG Holman L Enama ME. Safety, Pharmacokinetics and Neutralization of the Broadly Neutralizing HIV-1 Human Monoclonal Antibody VRC01 in Healthy Adults. Clin Exp Immunol (2015) 182(3):289–301. 10.1111/cei.12692 26332605 PMC4636891

[7] Mayer KH Seaton KE Huang Y Grunenberg N Isaacs A Allen M. Safety, Pharmacokinetics, and Immunological Activities of Multiple Intravenous or Subcutaneous Doses of an Anti-HIV Monoclonal Antibody, VRC01, Administered to HIV-Uninfected Adults: Results of a Phase 1 Randomized Trial. PloS Med (2017) 14(11):e1002435. 10.1371/journal.pmed.1002435 29136037 PMC5685476

[8] Huang Y Naidoo L Zhang L Carpp LN Rudnicki E Randhawa A. Pharmacokinetics and Predicted Neutralisation Coverage of VRC01 in HIV-Uninfected Participants of the Antibody Mediated Prevention (AMP) Trials. EBioMedicine (2021) 64:103203. 10.1016/j.ebiom.2020.103203 33493795 PMC7841500

[9] Corey L Gilbert PB Juraska M Montefiori DC Morris L Karuna ST. Two Randomized Trials of Neutralizing Antibodies to Prevent HIV-1 Acquisition. New Engl J Med (2021) 384(11):1003–14. 10.1056/NEJMoa2031738 PMC8189692 33730454

[10] Edupuganti S Mgodi N Karuna ST Andrew P Rudnicki E Kochar N. Feasibility and Successful Enrollment in a Proof-Of-Concept HIV Prevention Trial of VRC01, A Broadly Neutralizing HIV-1 Monoclonal Antibody. JAIDS J Acquired Immune Deficiency Syndromes (2021) 87(1):671–79. Publish Ahead of Print. 10.1097/QAI.0000000000002639 PMC8397466 33587505

[11] Caskey M Klein F Lorenzi JCC Seaman MS West AP Buckley N. Viraemia Suppressed in HIV-1-Infected Humans by Broadly Neutralizing Antibody 3BNC117. Nature (2015) 522(7557):487–91. 10.1038/nature14411 PMC4890714 25855300

[12] Bar-On Y Gruell H Schoofs T Pai JA Nogueira L Butler AL. Safety and Antiviral Activity of Combination HIV-1 Broadly Neutralizing Antibodies in Viremic Individuals. Nat Med (2018) 24(11):1701–7. 10.1038/s41591-018-0186-4 PMC6221973 30258217

[13] Mendoza P Gruell H Nogueira L Pai JA Butler AL Millard K. Combination Therapy With Anti-HIV-1 Antibodies Maintains Viral Suppression. Nature (2018) 561(7724):479–84. 10.1038/s41586-018-0531-2 PMC6166473 30258136

[14] Li Y Migueles SA Welcher B Svehla K Phogat A Louder MK. Broad HIV-1 Neutralization Mediated by CD4-Binding Site Antibodies. Nat Med (2007) 13(9):1032–4. 10.1038/nm1624 PMC2584972 17721546

[15] Wu X Yang Z-Y Li Y Hogerkorp C-M Schief WR Seaman MS. Rational Design of Envelope Identifies Broadly Neutralizing Human Monoclonal Antibodies to HIV-1. Science (2010) 329(5993):856. 10.1126/science.1187659 20616233 PMC2965066

[16] Walker LM Huber M Doores KJ Falkowska E Pejchal R Julien J-P. Broad Neutralization Coverage of HIV by Multiple Highly Potent Antibodies. Nature (2011) 477(7365):466–70. 10.1038/nature10373 PMC3393110 21849977

[17] Rosenberg YJ Montefiori DC LaBranche CC Lewis MG Sack M Lees JP. Protection Against SHIV Challenge by Subcutaneous Administration of the Plant-Derived PGT121 Broadly Neutralizing Antibody in Macaques. PloS One (2016) 11(3):e0152760. 10.1371/journal.pone.0152760 27031108 PMC4816452

[18] Sok D van Gils MJ Pauthner M Julien J-P Saye-Francisco KL Hsueh J. Recombinant HIV Envelope Trimer Selects for Quaternary-Dependent Antibodies Targeting the Trimer Apex. Proc Natl Acad Sci (2014) 111(49):17624. 10.1073/pnas.1415789111 25422458 PMC4267403

[19] Grobben M Stuart RAL van Gils MJ. The Potential of Engineered Antibodies for HIV-1 Therapy and Cure. Curr Opin Virol (2019) 38:70–80. 10.1016/j.coviro.2019.07.007 31421319

[20] Rudicell RS Kwon YD Ko S-Y Pegu A Louder MK Georgiev IS. Enhanced Potency of a Broadly Neutralizing HIV-1 Antibody In Vitro Improves Protection Against Lentiviral Infection In Vivo. J Virol (2014) 88(21):12669. 10.1128/JVI.02213-14 25142607 PMC4248941

[21] Tomaras GD Yates NL Liu P Qin L Fouda GG Chavez LL. Initial B-Cell Responses to Transmitted Human Immunodeficiency Virus Type 1: Virion-Binding Immunoglobulin M (IgM) and IgG Antibodies Followed by Plasma Anti-Gp41 Antibodies With Ineffective Control of Initial Viremia. J Virol (2008) 82(24):12449. 10.1128/JVI.01708-08 18842730 PMC2593361

[22] Fouda GG Yates NL Pollara J Shen X Overman GR Mahlokozera T. HIV-Specific Functional Antibody Responses in Breast Milk Mirror Those in Plasma and Are Primarily Mediated by IgG Antibodies. J Virol (2011) 85(18):9555. 10.1128/JVI.05174-11 21734046 PMC3165739

[23] Yates NL Lucas JT Nolen TL Vandergrift NA Soderberg KA Seaton KE. Multiple HIV-1-Specific IgG3 Responses Decline During Acute HIV-1: Implications for Detection of Incident HIV Infection. AIDS (2011) 25(17) 2089–97. 10.1097/QAD.0b013e32834b348e PMC3667583 21832938

[24] Liu P Overman RG Yates NL Alam SM Vandergrift N Chen Y. Dynamic Antibody Specificities and Virion Concentrations in Circulating Immune Complexes in Acute to Chronic HIV-1 Infection. J Virol (2011) 85(21):11196. 10.1128/JVI.05601-11 21865397 PMC3194959

[25] Eckels J Nathe C Nelson EK Shoemaker SG Nostrand EV Yates NL. Quality Control, Analysis and Secure Sharing of Luminex^®^ Immunoassay Data Using the Open Source LabKey Server Platform. BMC Bioinf (2013) 14(1):145. 10.1186/1471-2105-14-145 PMC3671158 23631706

[26] Bioanalytical Method Validation FDA. Guidance for Industry. Www.Fda.Com: Food and Drug Administration, Centre for Drug Evaluation and Research (CDER). Available at: https://www.fda.gov/files/drugs/published/Bioanalytical-Method-Validation-Guidance-for-Industry.pdf.

[27] ICH. Validation of Analytical Procedures: Text and Methodology. Q2(R1): ICH Harmonised Tripartite Guidline (2005). Available at: https://database.ich.org/sites/default/files/Q2_R1:Guideline.pdf.

[28] Armbruster D Pry T. Limit of Blank, Limit of Detection and Limit of Quantitation. Clin biochemist Reviews/Australian Assoc Clin Biochemists (2008) 29(Suppl 1):S49–52. PMC2556583 18852857

[29] Zhang J Li W Roskos LK Yang H. Immunogenicity Assay Cut Point Determination Using Nonparametric Tolerance Limit. J Immunol Methods (2017) 442:29–34. 10.1016/j.jim.2017.01.001 28063769

[30] Shankar G Devanarayan V Amaravadi L Barrett YC Bowsher R Finco-Kent D. Recommendations for the Validation of Immunoassays Used for Detection of Host Antibodies Against Biotechnology Products. J Pharm Biomed Anal (2008) 48(5):1267–81. 10.1016/j.jpba.2008.09.020 18993008

[31] Haraya K Tachibana T Igawa T. Improvement of Pharmacokinetic Properties of Therapeutic Antibodies by Antibody Engineering. Drug Metab Pharmacokinetics (2019) 34(1):25–41. 10.1016/j.dmpk.2018.10.003 30472066

[32] Glassman PM Balthasar JP. Physiologically-Based Modeling of Monoclonal Antibody Pharmacokinetics in Drug Discovery and Development. Drug Metab Pharmacokinetics (2019) 34(1):3–13. 10.1016/j.dmpk.2018.11.002 PMC6378116 30522890

[33] Liu Q Lai Y-T Zhang P Louder MK Pegu A Rawi R. Improvement of Antibody Functionality by Structure-Guided Paratope Engraftment. Nat Commun (2019) 10(1):721. 10.1038/s41467-019-08658-4 30760721 PMC6374468

[34] Gaudinski MR Houser KV Doria-Rose NA Chen GL Rothwell RSS Berkowitz N. Safety and Pharmacokinetics of Broadly Neutralising Human Monoclonal Antibody VRC07-523LS in Healthy Adults: A Phase 1 Dose-Escalation Clinical Trial. Lancet HIV (2019) 6(10):e667–79. 10.1016/S2352-3018(19)30181-X PMC11100866 31473167

[35] Julg B Tartaglia LJ Keele BF Wagh K Pegu A Sok D. Broadly Neutralizing Antibodies Targeting the HIV-1 Envelope V2 Apex Confer Protection Against a Clade C SHIV Challenge. Sci Transl Med (2017) 9(406):eaal1321. 10.1126/scitranslmed.aal1321 28878010 PMC5755978

