# Supplementary material for: Validation of a Triplex Pharmacokinetic Assay for Simultaneous Quantitation of HIV-1 Broadly Neutralizing Antibodies PGT121, PGDM1400, and VRC07-523-LS
Source: Front Immunol. 2021 Aug 24;12:709994. doi: 10.3389/fimmu.2021.709994 (PMC8422903; doi:10.3389/fimmu.2021.709994)
Supplement: Supplementary file 1 [file DataSheet_1.docx]

Supplementary Material

**
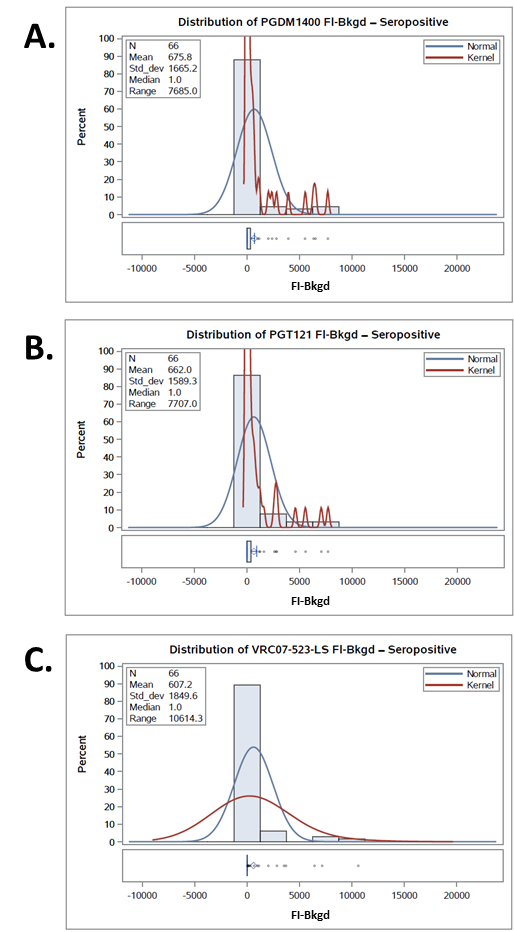
**

**Supplementary Figure 1. Non-specific background activity of HIV-1 seropositive human serum.** 66 HIV-1 seropositive samples were tested at 1:50 in the PGDM1400/PGT121/VRC07-523-LS PK BAMA, and the distribution of binding to the PGDM1400 anti-ID (A), PGT121 anti-ID (B), and VRC07-523-LS anti-ID (C) was plotted. Normal and kernel density estimates are used to visualize the data distribution. Normal shows the fit of a normal distribution to the FI-Bkgd measurements, and a superimposed fitted density curve on the histogram. Kernel density estimates on histograms help smoothen the data distribution, and help visualize the non-normal data distribution and patterns. All 3 kernel density graphs were generated using the same SAS code with the same x-axis scale. Since VRC07-523-LS had a wider FI-Bkgd range, kernel graphs for PGT121 and PGDM1400 were plotted onto the same axis scale for comparison.

**Supplementary Figure 2. Positivity of the PGDM1400, PGT121 and VRC07-523-LS Triplex PK in HIV-1 seropositive human serum.** 17 seropositive samples from subjects that were co-infused with PGDM1400 and PGT121 were spiked with VRC07-523-LS in similar concentrations reported for PDGM1400 and PGT121. A scatter plot of the observed and expected concentration of PGDM1400, PGT121, and VRC07-523-LS, respectively, is shown. The dotted line represents an intercept =0 and slope=1 line. Percentages of samples where the observed concentration in the Triplex PK are within 2-fold of the expected concentration are shown as well as the number of samples that met this criteria over the total number of samples with detectable antibody. Green dots show samples with observed concentrations within 2-fold of the expected concentrations, and red dots show the samples with more than 2-fold difference between observed and expected concentrations.

**Supplementary Figure 3. Accuracy of Standard Curves and Spiked Sample Recovery.** PGDM1400 IgG (A), PGT121 IgG (C) and VRC07-523-LS IgG (E) mAbs were titrated in diluent (red), in pooled 1:100 diluted HIV-1 seropositive human serum. PGDM1400 IgG (B), PGT121 IgG (D) and VRC07-523-LS IgG (F) mAbs were spiked in pooled 1:100 diluted seropositive serum at 5 different concentrations. Observed concentration for each point in the curve was calculated and used to determine accuracy of recovery of each mAb. The dotted lines denote the upper (130% of the expected concentration) and lower (70% of the expected concentration) limits of acceptable percent recovery.

**Supplementary Figure 4. Accuracy of the PGDM1400/PGT121/VRC07-523-LS PK BAMA standard curves in diluent and HIV-1 seropositive human serum.** PGDM1400 (A), PGT121 (B) and VRC07-523-LS (C) were co-titrated in assay diluent and in 1:100 HIV-1 seropositive serum diluted in assay diluent. 5PL EC50 values of each mAb fell within 3 standard deviations of the historical mean (solid black line).

**Supplementary Table 1. Limit of Detection and Quantification in HIV-1 seropositive human serum.** LOD, LLOQ and ULOQ as well as the corresponding physiological concentrations observed in the PGDM1400/PGT121/VRC07-523-LS Triplex PK for HIV-1 seropositive human serum.
